# The genome sequence of *Heligmosomum mixtum* Schulz, 1929 (Rhabditida: Heligmosomidae)

**DOI:** 10.12688/wellcomeopenres.26318.1

**Published:** 2026-04-17

**Authors:** Abosede Olarewaju, Agnieszka Kloch, Lewis Stevens, Manuela Kieninger, Michael Paulini, Wellcome Sanger Institute Tree of Life Management, Samples and Laboratory team, Wellcome Sanger Institute Scientific Operations: Sequencing Operations, Wellcome Sanger Institute Tree of Life Core Informatics team, Tree of Life Core Informatics collective, Mark L. Blaxter

**Affiliations:** 1 Faculty of Biology University of Warsaw, Warsaw, Poland; 2 Tree of Life Programme, Wellcome Sanger Institute, Hinxton, England, UK

**Keywords:** Heligmosomum mixtum, genome sequence, chromosomal, Rhabditida

## Abstract

We present a genome assembly of *Heligmosomum mixtum* (Nematoda; Chromadorea; Rhabditida; Heligmosomidae). The assembly consists of two haplotypes with total lengths of 741.91 megabases and 754.62 megabases. Most of haplotype 1 (98.33%) is scaffolded into 6 chromosomal pseudomolecules, including the X sex chromosome. Haplotype 2 was assembled to scaffold level. The mitochondrial genome has been assembled, with a length of 13.53 kilobases.

## Species taxonomy

Eukaryota; Opisthokonta; Metazoa; Eumetazoa; Bilateria; Protostomia; Ecdysozoa; Nematoda; Chromadorea; Rhabditida; Rhabditina; Rhabditomorpha; Strongyloidea; Heligmosomidae; *Heligmosomum*; *Heligmosomum mixtum* Schulz, 1929 (NCBI:txid375988).

## Background

*Heligmosomum mixtum* is a common gastrointestinal parasite of bank voles ( *Clethrionomys* = *Myodes glareolus* Schreber, 1780) and is widely used in parasitology and ecology ( Bajer *et al.*, 2005; Behnke *et al.*, 2001, 2009; Kloch *et al.*, 2010). More recently, it has been a focus of eco-immunology and phylogeography research ( McManus *et al.*, 2021; Sakka *et al.*, 2014). The genus *Heligmosomum* comprises 12 nominal species, which are nested within the clades of the paraphyletic *Heligmosomoides* genus ( Alnaqeb *et al.*, 2022; Harris *et al.*, 2015; Zaleśny *et al.*, 2014). These gastrointestinal parasites reside in the small intestines, particularly the duodenum, of voles and mice distributed throughout the Palearctic and Nearctic regions, i.e., the Holarctic region ( Durette-Desset, 1985; Harris *et al.*, 2015). In continental Europe, these parasites are typically the dominant species found, except in the eastern region where the species from the *Heligmosomoides* genus can be predominant ( Haukisalmi & Henttonen, 2000). While *M. glareolus* is the main host of *H. mixtum* ( Butmyrin *et al.*, 2005), they have been found in other rodent species such as *Clethrionomys rutilus*, *Microtus arvalis*, *Microtus agrestis*, *Microtus socialis*, and *Meriones persicus* ( Grikienię, 2005; Haukisalmi *et al.*, 1996; Kia *et al.*, 2010), as well as *Apodemus flavicollis* ( Mažeika *et al.*, 2003).

*H. mixtum* are dioecious parasites, exhibit a direct life cycle, and inhabit the intestinal lumen of the duodenum ( Asakawa, 1987; Bryant, 1973; Sukhdeo *et al.*, 1984), primarily feeding on gut content ( Haukisalmi & Henttonen, 1993). They reproduce sexually with three free-living larval stages and a 4th, parasitic larval stage that resides in the intestinal wall ( Asakawa, 1987). Infection occurs when hosts ingest food or water contaminated with the infective larval stage (L3) of *H. mixtum.* The life cycle begins when the adult females produce eggs, which are expelled into the environment through the host’s faeces. The eggs hatch into the first-stage larvae (L1). These larvae undergo two additional moults, developing into second-stage (L2) and third-stage larvae (L3), which are free-living and can survive in various environmental conditions until a suitable host ingests them. Once ingested by a rodent, the L3 larvae migrate through the mucosa to the intestines, shedding their sheath and growing to L4 and L5 before developing into adult worms ( Asakawa, 1987; Ehrenford, 1954). Adult *H. mixtum* reside in the intestinal lumen, where they reproduce sexually, completing the cycle by producing new eggs that are excreted with the host’s faeces. Larval migration through the intestinal mucosa during various life stages can cause mild inflammation, as observed with *Heligmosomum costellatum* in common voles ( Janova *et al.*, 2010), a close relative of the bank vole. While specific details regarding the lifespan and mating behaviours of *H. mixtum* are limited ( Kloch *et al.*, 2015), it is believed that many helminth parasites, including *H. mixtum*, exhibit relatively long lifespans comparable to their rodent hosts ( Behnke *et al.*, 2009; Haukisalmi *et al.*, 1996), potentially employing immunosuppression strategies to persist within hosts ( Behnke *et al.*, 2009; Hewitson *et al.*, 2011; Maizels *et al.*, 2012).

Sakka *et al.* (2014) showed that the genetic analysis of the cytochrome b gene (cytb) in mitochondrial DNA reveals a weak phylogeographical structure for *H. mixtum* compared to its primary host, *M. glareolus*, along with low genetic diversity relative to its counterpart *Heligmosomoides polygyrus*, suggesting a rapidly expanding population with a demographic history characterised by a unimodal distribution linked to past bottlenecks during the last glacial maximum.

## Methods

### Sample acquisition

We collected *H. mixtum* from *M. glareolus* captured in Okuniew, Warsaw, in central Poland (latitude 52.279, longitude 21.32) in Autumn 2023. Voles were caught live in locally constructed wooden traps, with a small metal internal platform triggered by a vole to release a metal door ( Bajer *et al.*, 2005; Behnke *et al.*, 2001). Wooden traps were set out at 20 m intervals in parallel lines, 10 m on either side of tracks running through the site and 2 traps were placed within 2–3 m of one another at each point ( Bajer *et al.*, 2005; Behnke *et al.*, 2001). The traps were baited with a mixture of wheat and peanut butter, as well as fruits like apples and carrots, to provide water. Captured voles were weighed (± 1 g), sexed, and euthanised following the European ethical standard (Directive 2010/63/EU) through anaesthetic overdose and cervical dislocation. The voles were sectioned, and their internal organs and intestinal tract were extracted and examined for intestinal parasites. Parasite species identification was carried out as described in Kloch *et al.* (2010). Briefly, in the laboratory, each intestine was divided into its anatomical sections (stomach, small intestine, caecum, and colon) and carefully examined under a microscope. All *H. mixtum* individuals were removed, counted, and snap-frozen, then stored at −80 °C until being transported to the Wellcome Sanger Institute for genome sequencing.

### Nucleic acid extraction

High molecular weight DNA was extracted from a frozen male adult of *Heligmosomum mixtum* (nxHelMixt5). DNA extraction was performed using a modified Monarch HMW DNA Extraction Kit ( Kieninger, 2025). A total of 40 ng of sheared DNA was used for library preparation.

### PacBio HiFi library preparation and sequencing

Library preparation and sequencing were performed at the WSI Scientific Operations core. Prior to library preparation, the DNA was fragmented to ~10 kb. Ultra-low-input (ULI) libraries were prepared using the PacBio SMRTbell ^®^ Express Template Prep Kit 2.0 and gDNA Sample Amplification Kit. Samples were normalised to 20 ng DNA. Single-strand overhang removal, DNA damage repair, and end-repair/A-tailing were performed according to the manufacturer’s instructions, followed by adapter ligation. A 0.85× pre-PCR clean-up was carried out with Promega ProNex beads.

The DNA was evenly divided into two aliquots for dual PCR (reactions A and B), both following the manufacturer’s protocol. A 0.85× post-PCR clean-up was performed with ProNex beads. DNA concentration was measured using a Qubit Fluorometer v4.0 (Thermo Fisher Scientific) with the Qubit HS Assay Kit, and fragment size was assessed on an Agilent Femto Pulse Automated Pulsed Field CE Instrument (Agilent Technologies) using the gDNA 55 kb BAC analysis kit. PCR reactions A and B were then pooled, ensuring a total mass of ≥500 ng in 47.4 μl.

The pooled sample underwent another round of DNA damage repair, end-repair/A-tailing, and hairpin adapter ligation. A 1× clean-up was performed with ProNex beads, followed by DNA quantification using the Qubit and fragment size analysis using the Agilent Femto Pulse. Size selection was performed on the Sage Sciences PippinHT system, with target fragment size determined by Femto Pulse analysis (typically 4–9 kb). Size-selected libraries were cleaned with 1.0× ProNex beads and normalised to 2 nM before sequencing.

The sample was sequenced on a Revio instrument (Pacific Biosciences). The prepared library was normalised to 2 nM, and 15 μL was used for making complexes. Primers were annealed and polymerases bound to generate circularised complexes, following the manufacturer’s instructions. Complexes were purified using 1.2X SMRTbell beads, then diluted to the Revio loading concentration (200–300 pM) and spiked with a Revio sequencing internal control. The sample was sequenced on a Revio 25 M SMRT cell. The SMRT Link software (Pacific Biosciences), a web-based workflow manager, was used to configure and monitor the run and to carry out primary and secondary data analysis.

### Hi-C

***Sample preparation and crosslinking***

Hi-C data were generated from the whole organism tissue of nxHelMixt1 using the Arima-HiC v2 kit (Arima Genomics). Tissue was finely ground using the Covaris cryoPREP Dry Pulverizer (Covaris), and then subjected to nuclei isolation. Nuclei were isolated using a modified protocol based on the Qiagen QProteome Cell Compartment Kit (Qiagen), in which only the Lysis and CE2 buffers were used, with QIAshredder spin columns. After isolation, nuclei were fixed using formaldehyde to a final concentration of 2% to crosslink the DNA. The crosslinked DNA was then digested and biotinylated according to the manufacturer’s instructions. A clean-up step was performed with SPRIselect beads before library preparation. DNA concentration was quantified using the Qubit Fluorometer v4.0 (Thermo Fisher Scientific) and the Qubit HS Assay Kit, following the manufacturer’s instructions.

***Hi-C library preparation and sequencing***

Biotinylated DNA constructs were fragmented using a Covaris E220 sonicator and size selected to 400–600 bp using SPRISelect beads. DNA was enriched with Arima-HiC v2 kit Enrichment beads. End repair, A-tailing, and adapter ligation were carried out with the NEBNext Ultra II DNA Library Prep Kit (New England Biolabs), following a modified protocol where library preparation occurs while DNA remains bound to the Enrichment beads. Library amplification was performed using KAPA HiFi HotStart mix and a custom Unique Dual Index (UDI) barcode set (Integrated DNA Technologies). Depending on sample concentration and biotinylation percentage determined at the crosslinking stage, libraries were amplified with 10–16 PCR cycles. Post-PCR clean-up was performed with SPRISelect beads. Libraries were quantified using the AccuClear Ultra High Sensitivity dsDNA Standards Assay Kit (Biotium) and a FLUOstar Omega plate reader (BMG Labtech).

Prior to sequencing, libraries were normalised to 10 ng/μL. Normalised libraries were quantified again to create equimolar and/or weighted 2.8 nM pools. Pool concentrations were checked using the Agilent 4200 TapeStation (Agilent) with High Sensitivity D500 reagents before sequencing. Sequencing was performed using paired-end 150 bp reads on the Illumina NovaSeq X.

### Genome assembly

Prior to assembly of the PacBio HiFi reads, a database of *k*-mer counts ( *k* = 31) was generated from the filtered reads using FastK. GenomeScope2 ( Ranallo-Benavidez *et al.*, 2020) was used to analyse the *k*-mer frequency distributions, providing estimates of genome size, heterozygosity, and repeat content.

The HiFi reads were assembled using Hifiasm in Hi-C phasing mode ( Cheng *et al.*, 2021, 2022), producing two haplotypes. Hi-C reads ( Rao *et al.*, 2014) were mapped to the primary contigs using bwa-mem2 ( Vasimuddin *et al.*, 2019). Contigs were further scaffolded with Hi-C data in YaHS ( Zhou *et al.*, 2023), using the --break option for handling potential misassemblies. The scaffolded assemblies were evaluated using Gfastats ( Formenti *et al.*, 2022), BUSCO ( Manni *et al.*, 2021) and MERQURY.FK ( Rhie *et al.*, 2020). The mitochondrial genome was assembled using MitoHiFi ( Uliano-Silva *et al.*, 2023).

### Assembly curation

The assembly was decontaminated using the Assembly Screen for Cobionts and Contaminants ( ASCC) pipeline. TreeVal was used to generate the flat files and maps for use in curation. Manual curation was conducted primarily in PretextView and HiGlass ( Kerpedjiev *et al.*, 2018). Scaffolds were visually inspected and corrected as described by Howe *et al*. (2021). Manual corrections included 227 breaks and 337 joins. This reduced the scaffold count by 30.5%, increased the scaffold N50 by 4.8%, and reduced the total assembly length by 0.8%. During curation, we observed that there is a haplotypic inversion on chromosome III (3–11.3 Mbp). The curation process is documented at https://gitlab.com/wtsi-grit/rapid-curation . PretextSnapshot was used to generate a Hi-C contact map of the final assembly.

### Assembly analysis and quality assessment

The distribution of Nigon elements, which represent the seven ancestral linkage groups in rhabditid nematodes ( Gonzalez de la Rosa *et al.*, 2021), was visualised by providing the ‘full_table.tsv’ file from the BUSCO nematoda_odb10 output to the R script vis_ALG.R, using a window size of 2 Mb.

The Merqury.FK tool ( Rhie *et al.*, 2020) was used to evaluate *k*-mer completeness and assembly quality for both haplotypes using the *k*-mer databases ( *k* = 31) computed prior to genome assembly. The analysis outputs included assembly QV scores and completeness statistics.

The genome was analysed using the BlobToolKit pipeline, a Nextflow implementation of the earlier Snakemake version ( Challis *et al.*, 2020). The pipeline aligns PacBio reads using minimap2 ( Li, 2018) and SAMtools ( Danecek *et al.*, 2021) to generate coverage tracks. It runs BUSCO ( Manni *et al.*, 2021) using lineages identified from NCBI Taxonomy ( Schoch *et al.*, 2020). For the three domain-level lineages, BUSCO genes are aligned to the UniProt Reference Proteomes database ( Bateman *et al.*, 2023) using DIAMOND blastp ( Buchfink *et al.*, 2021). The genome is divided into chunks based on the density of BUSCO genes from the closest taxonomic lineage, and each chunk is aligned to the UniProt Reference Proteomes database with DIAMOND blastx. Sequences without hits are chunked using seqtk and aligned to the NT database with blastn ( Altschul *et al.*, 1990). The BlobToolKit suite consolidates all outputs into a blobdir for visualisation. The BlobToolKit pipeline was developed using nf-core tooling ( Ewels *et al.*, 2020) and MultiQC ( Ewels *et al.*, 2016), with package management via Conda and Bioconda ( Grüning *et al.*, 2018), and containerisation through Docker ( Merkel, 2014) and Singularity ( Kurtzer *et al.*, 2017).

## Genome sequence report

### Sequence data

**Figure 1.**
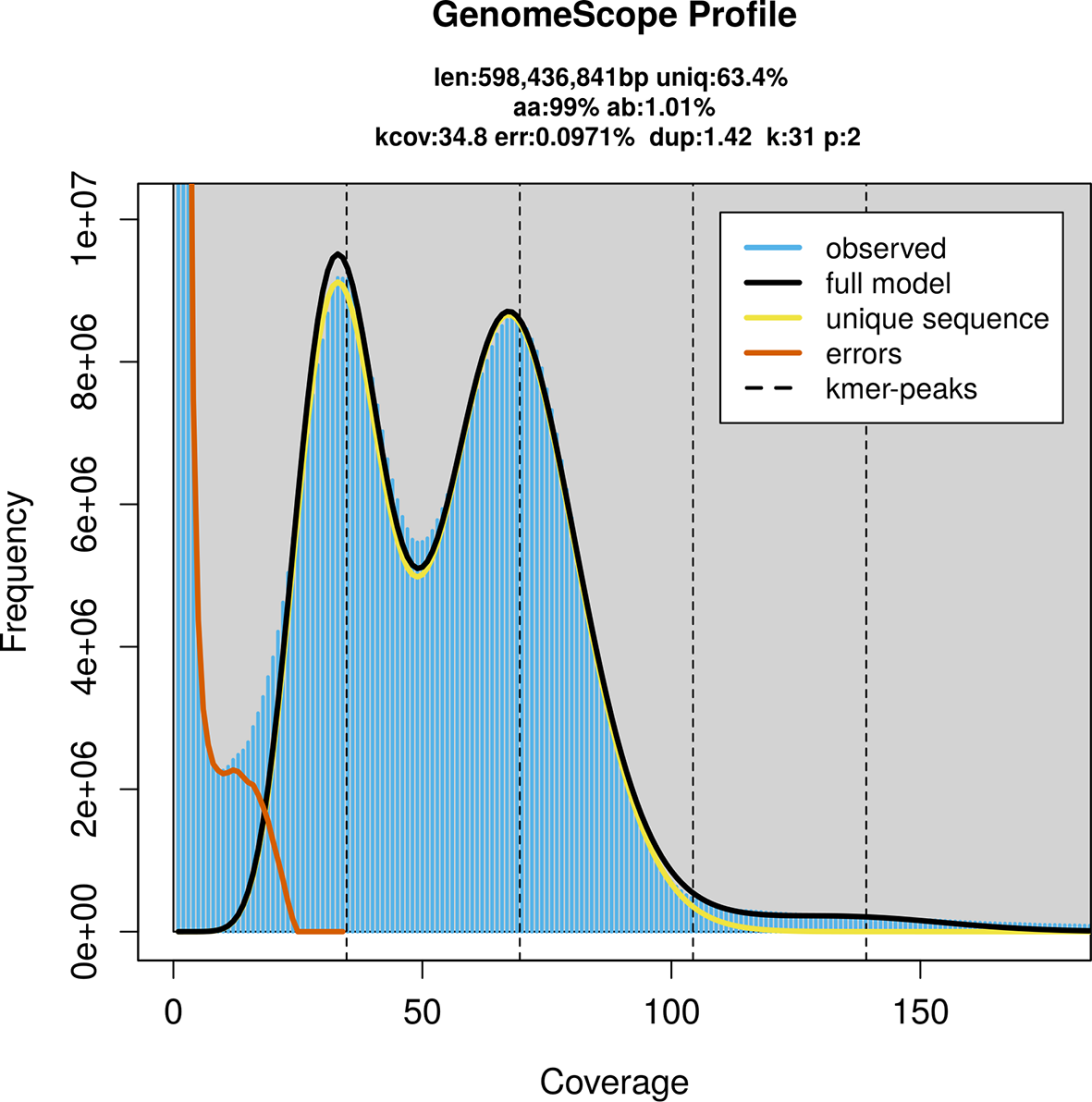
Frequency distribution of *k*-mers generated using GenomeScope2. The plot shows observed and modelled *k*-mer spectra, providing estimates of genome size, heterozygosity, and repeat content based on unassembled sequencing reads.

The genome of a specimen of *Heligmosomum mixtum* was sequenced using Pacific Biosciences single-molecule HiFi long reads, generating 44.43 Gb (gigabases) from 3.75 million reads, which were used to assemble the genome. GenomeScope2.0 analysis estimated the haploid genome size at 599.27 Mb, with a heterozygosity of 1.01% and repeat content of 36.60% ( Figure 1). These estimates guided expectations for the assembly. Based on the estimated genome size, the sequencing data provided approximately 70× coverage. Hi-C sequencing produced 116.37 Gb from 385.33 million reads, which were used to scaffold the assembly. Table 1 summarises the specimen and sequencing details.

**Table 1.** Specimen and sequencing data for BioProject PRJEB83168.

Platform	PacBio HiFi	Hi-C
**ToLID**	nxHelMixt5	nxHelMixt1
**Specimen ID**	SAN20002475	SAN20002477
**BioSample (source individual)**	SAMEA115287570	SAMEA115287566
**BioSample (tissue)**	SAMEA115287576	SAMEA115287572
**Tissue**	whole organism	whole organism
**Instrument**	Revio	Illumina NovaSeq X
**Run accessions**	ERR14048106	ERR14056209
**Read count total**	3.75 million	385.33 million
**Base count total**	44.43 Gb	116.37 Gb

### Assembly statistics

The genome was assembled into two haplotypes using Hi-C phasing. Haplotype 1 was curated to chromosome level, while haplotype 2 was assembled to scaffold level. The final assembly has a total length of 741.91 Mb in 197 scaffolds, with 2 594 gaps, and a scaffold N50 of 127.18 Mb ( Table 2).

**Table 2.** Genome assembly statistics.

Genome assembly	Haplotype 1	Haplotype 2
**Assembly name**	nxHelMixt5.hap1.1	nxHelMixt5.hap2.1
**Assembly accession**	GCA_965643505.1	GCA_965643595.1
**Assembly level**	chromosome	scaffold
**Span (Mb)**	741.91	754.62
**Number of chromosomes**	6	-
**Number of contigs**	2 791	15 381
**Contig N50**	0.5 Mb	0.11 Mb
**Number of scaffolds**	197	10 392
**Scaffold N50**	127.18 Mb	95.46 Mb
**Longest scaffold length (Mb)**	130.85	-
**Sex chromosomes**	X	-
**Organelles**	Mitochondrion: 13.53 kb	-

**Figure 2.**
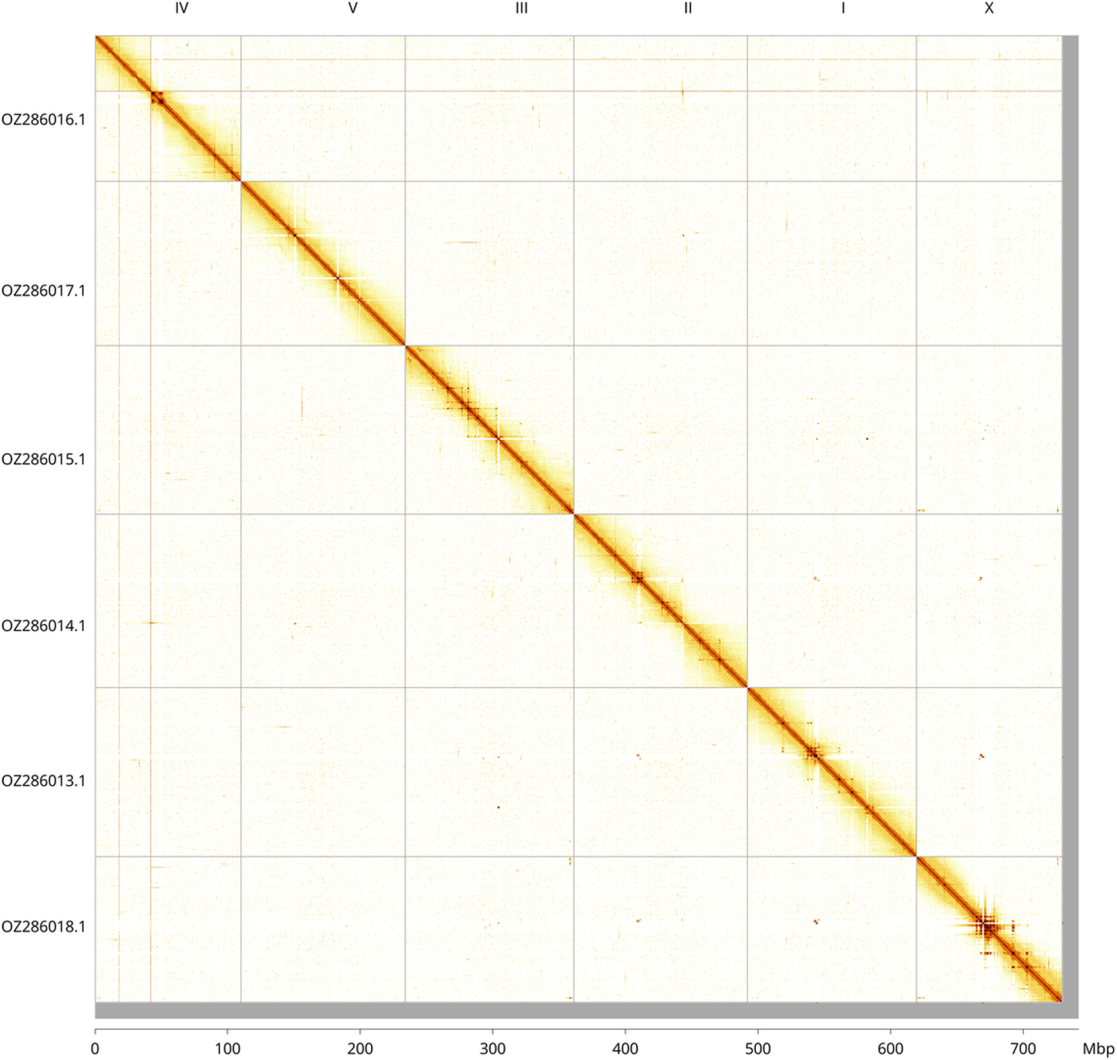
Hi-C contact map of the *Heligmosomum mixtum* genome assembly. Assembled chromosomes are shown in order of size and labelled along the axes, with a megabase scale shown below. The plot was generated using PretextSnapshot.

Most of the assembly sequence (98.33%) was assigned to 6 chromosomal-level scaffolds, representing 5 autosomes and the X sex chromosome. These chromosome-level scaffolds, confirmed by Hi-C data, are named according to synteny ( Figure 2; Table 3).

**Table 3.** Chromosomal pseudomolecules in the haplotype 1 genome assembly of *Heligmosomum mixtum* nxHelMixt5.

INSDC accession	Molecule	Length (Mb)	GC%
OZ286018.1	X	109.94	46.50
OZ286013.1	I	110.24	46.50
OZ286014.1	II	123.88	45.50
OZ286015.1	III	127.18	46.50
OZ286016.1	IV	130.85	46
OZ286017.1	V	127.43	46

The mitochondrial genome was also assembled. This sequence is included as a contig in the multifasta file of the genome submission and as a standalone record.

**Figure 3.**
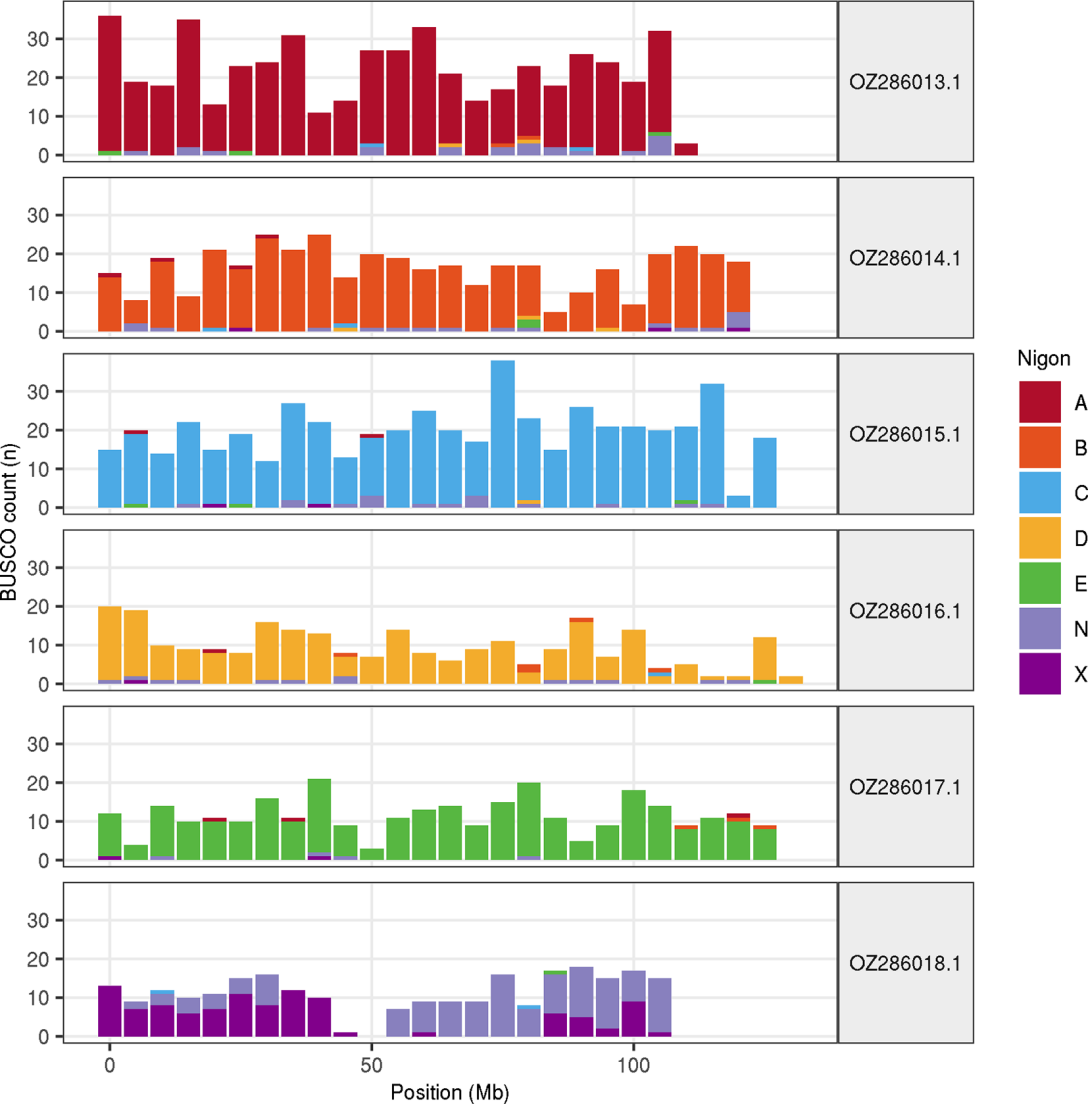
Nigon element painting of chromosomes in the nxHelMixt5.hap1.1 assembly of *Heligmosomum mixtum.* Counts of Benchmarking using Single Copy Orthologues (BUSCO) loci in 500 kb windows are coloured by their allocation to the seven Nigon elements (A-E, N, X).

Chromosome painting with Nigon elements illustrates the distribution of orthologues along chromosomes and highlights patterns of chromosomal evolution relative to ancestral linkage groups ( Figure 3).

### Assembly quality metrics

**Figure 4.**
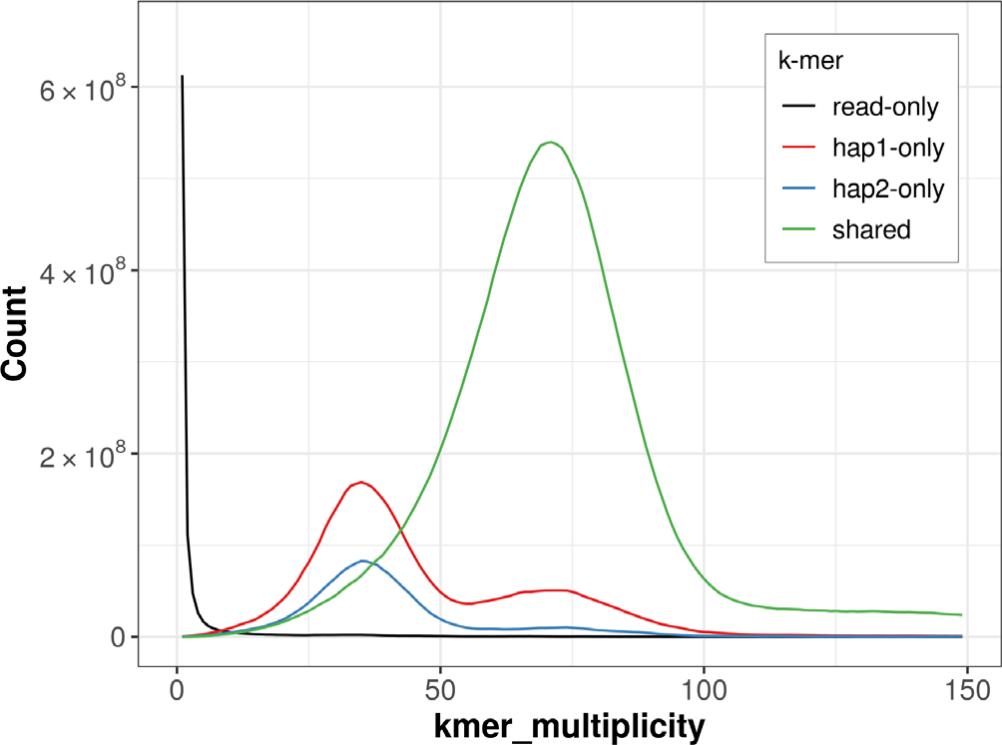
Evaluation of *k*-mer completeness using MerquryFK. This plot illustrates the recovery of *k*-mers from the original read data in the final assemblies. The horizontal axis represents *k*-mer multiplicity, and the vertical axis shows the number of *k*-mers. The black curve represents *k*-mers that appear in the reads but are not assembled. The green curve corresponds to *k*-mers shared by both haplotypes, and the red and blue curves show *k*-mers found only in one of the haplotypes.

For haplotype 1, the estimated QV is 58.2, and for haplotype 2, 57.0. When the two haplotypes are combined, the assembly achieves an estimated QV of 57.5. The *k*-mer completeness is 88.23% for haplotype 1, 73.52% for haplotype 2, and 99.23% for the combined haplotypes ( Figure 4).

**Figure 5.**
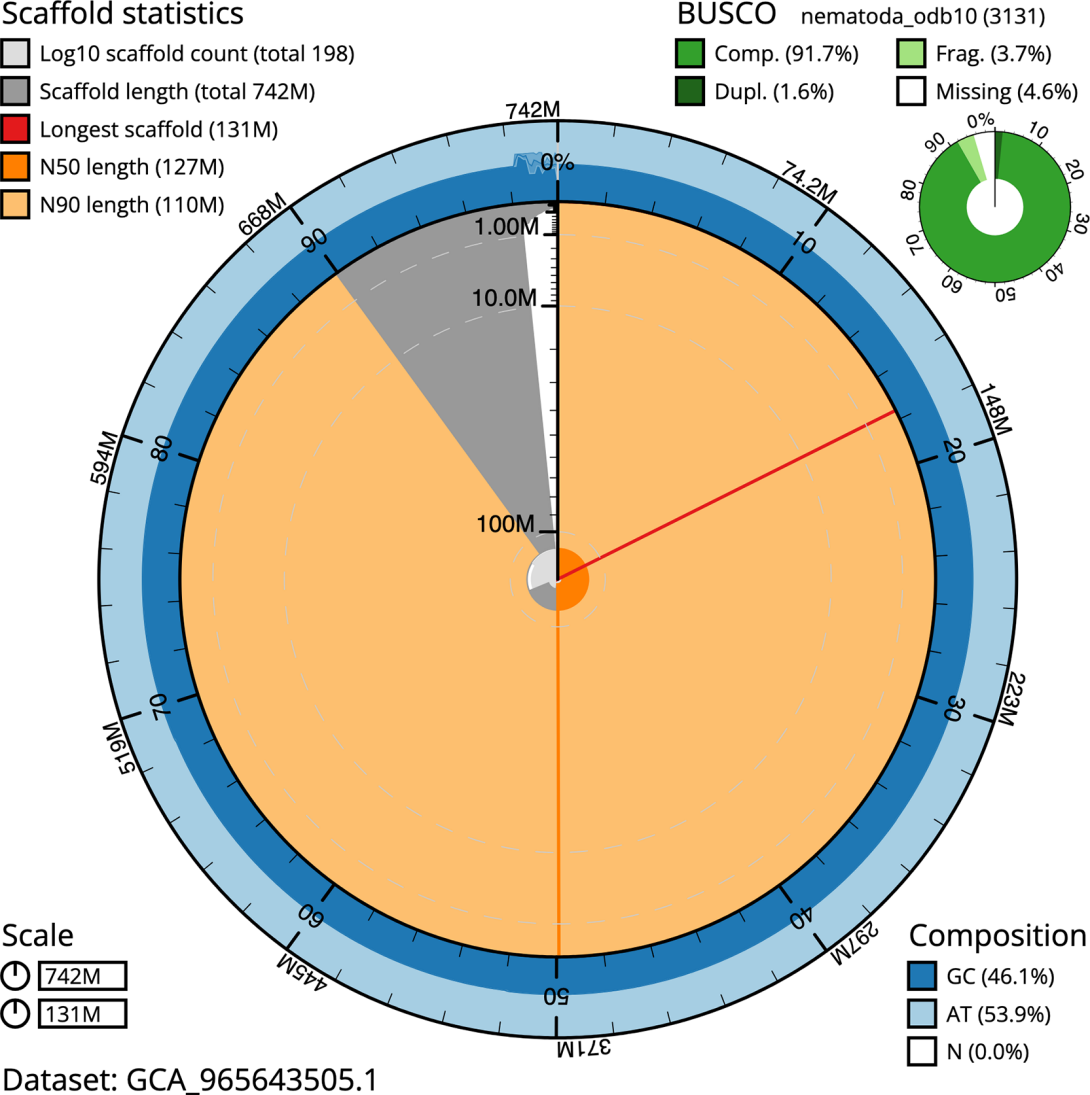
Assembly metrics for nxHelMixt5.hap1.1. The BlobToolKit snail plot provides an overview of assembly metrics and BUSCO gene completeness. The circumference represents the length of the whole genome sequence, and the main plot is divided into 1,000 bins around the circumference. The outermost blue tracks display the distribution of GC, AT, and N percentages across the bins. Scaffolds are arranged clockwise from longest to shortest and are depicted in dark grey. The longest scaffold is indicated by the red arc, and the deeper orange and pale orange arcs represent the N50 and N90 lengths. A light grey spiral at the centre shows the cumulative scaffold count on a logarithmic scale. A summary of complete, fragmented, duplicated, and missing BUSCO genes in the set is presented at the top right. An interactive version of this figure can be accessed on the BlobToolKit viewer.

**Figure 6.**
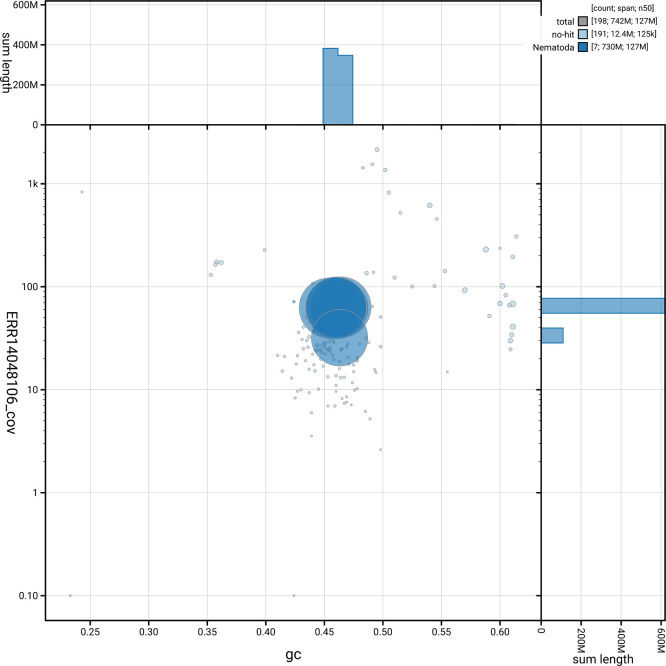
BlobToolKit GC-coverage plot for nxHelMixt5.hap1.1. Blob plot showing sequence coverage (vertical axis) and GC content (horizontal axis). The circles represent scaffolds, with the size proportional to scaffold length and the colour representing phylum membership. The histograms along the axes display the total length of sequences distributed across different levels of coverage and GC content. An interactive version of this figure is available on the BlobToolKit viewer.

BUSCO analysis using the nematoda_odb10 reference set ( *n* = 3 131) identified 91.7% of the expected gene set (single = 90.1%, duplicated = 1.6%) for haplotype 1. The snail plot in Figure 5 summarises the scaffold length distribution and other assembly statistics for haplotype 1. The blob plot in Figure 6 shows the distribution of scaffolds by GC proportion and coverage for haplotype 1.

Table 4 lists the assembly metric benchmarks adapted from Rhie *et al.* (2021) and the Earth BioGenome Project Report on Assembly Standards September 2024. The EBP metric calculated for the haplotype 1 is **5.C.Q58**.

**Table 4.** Earth Biogenome Project summary metrics for the *Heligmosomum mixtum* assembly.

Measure	Value	Benchmark
EBP summary (haplotype 1)	5.C.Q58	6.C.Q40
Contig N50 length	0.50 Mb	≥ 1 Mb
Scaffold N50 length	127.18 Mb	= chromosome N50
Consensus quality (QV)	Haplotype 1: 58.2; haplotype 2: 57.0; combined: 57.5	≥ 40
*k*-mer completeness	Haplotype 1: 88.23%; Haplotype 2: 73.52%; combined: 99.23%	≥ 95%
BUSCO	C:91.7% [S:90.1%, D:1.6%], F:3.7%, M:4.6%, n:3 131	S > 90%; D < 5%
Percentage of assembly assigned to chromosomes	98.33%	≥ 90%

*Notes:* The EBP summary uses log10(Contig N50); chromosome-level (C) or log10(Scaffold N50); Q (Merqury QV). BUSCO: C = complete; S = single-copy; D = duplicated; F = fragmented; M = missing; n = orthologues.

**Table 5.** Software versions and sources.

Software	Version	Source
BLAST	2.14.0	ftp://ftp.ncbi.nlm.nih.gov/blast/executables/blast+/
BlobToolKit	4.4.6	https://github.com/blobtoolkit/blobtoolkit
BUSCO	5.8.3	https://gitlab.com/ezlab/busco
bwa-mem2	2.2.1	https://github.com/bwa-mem2/bwa-mem2
DIAMOND	2.1.8	https://github.com/bbuchfink/diamond
fasta_windows	0.2.4	https://github.com/tolkit/fasta_windows
FastK	1.1	https://github.com/thegenemyers/FASTK
GenomeScope2.0	2.0.1	https://github.com/tbenavi1/genomescope2.0
Gfastats	1.3.6	https://github.com/vgl-hub/gfastats
Hifiasm	0.19.8-r603	https://github.com/chhylp123/hifiasm
HiGlass	1.13.4	https://github.com/higlass/higlass
MerquryFK	1.1.2	https://github.com/thegenemyers/MERQURY.FK
Minimap2	2.28-r1209	https://github.com/lh3/minimap2
MitoHiFi	3	https://github.com/marcelauliano/MitoHiFi
MultiQC	1.14; 1.17 and 1.18	https://github.com/MultiQC/MultiQC
Nextflow	24.10.4	https://github.com/nextflow-io/nextflow
PretextSnapshot	0.0.5	https://github.com/sanger-tol/PretextSnapshot
PretextView	1.0.3	https://github.com/sanger-tol/PretextView
samtools	1.21	https://github.com/samtools/samtools
sanger-tol/ascc	0.1.0	https://github.com/sanger-tol/ascc
sanger-tol/blobtoolkit	v0.8.0	https://github.com/sanger-tol/blobtoolkit
sanger-tol/curationpretext	1.4.2	https://github.com/sanger-tol/curationpretext
Seqtk	1.3	https://github.com/lh3/seqtk
Singularity	3.9.0	https://github.com/sylabs/singularity
TreeVal	1.4.0	https://github.com/sanger-tol/treeval
YaHS	1.2.2	https://github.com/c-zhou/yahs

## Author information

• Members of the Wellcome Sanger Institute Tree of Life Management, Samples and Laboratory team
• Members of Wellcome Sanger Institute Scientific Operations – Sequencing Operations
• Members of the Wellcome Sanger Institute Tree of Life Core Informatics team
• Members of the Tree of Life Core Informatics collective

## Wellcome Sanger Institute – Legal and Governance

The materials that have contributed to this genome note have been supplied by a Tree of Life collaborator. The Wellcome Sanger Institute employs a process whereby due diligence is carried out proportionate to the nature of the materials themselves, and the circumstances under which they have been/are to be collected and provided for use. The purpose of this is to address and mitigate any potential legal and/or ethical implications of receipt and use of the materials as part of the research project, and to ensure that in doing so, we align with best practice wherever possible. The overarching areas of consideration are:

• Ethical review of provenance and sourcing of the material.
• Legality of collection, transfer and use (national and international).

Each transfer of samples is undertaken according to a Research Collaboration Agreement or Material Transfer Agreement entered into by the Tree of Life collaborator, Genome Research Limited (operating as the Wellcome Sanger Institute), and in some circumstances, other Tree of Life collaborators.

## Data Availability

European Nucleotide Archive: Heligmosomum mixtum. Accession number PRJEB83168. The genome sequence is released openly for reuse. The *Heligmosomum mixtum* genome sequencing initiative is part of the Sanger Institute Tree of Life Programme (PRJEB43745) and 959NG (PRJEB81973). All raw sequence data and the assembly have been deposited in INSDC databases. The genome will be annotated using available RNA-Seq data and presented through the Ensembl pipeline at the European Bioinformatics Institute. Raw data and assembly accession identifiers are reported in Tables 1 and 2. Pipelines used for genome assembly at the WSI Tree of Life are available at https://pipelines.tol.sanger.ac.uk/pipelines. Table 5 lists software versions used in this study.
