# Supplementary material for: The genome sequence of Heligmosomum mixtum Schulz, 1929 (Rhabditida: Heligmosomidae)
Source: Wellcome Open Res. 2026 Apr 17;11:225. [Version 1] doi: 10.12688/wellcomeopenres.26318.1 (PMC13173282; doi:10.12688/wellcomeopenres.26318.1)
Supplement: Peer review history

# Reviewer report: Reviewer response for version 1

**DOI:** 10.21956/wellcomeopenres.28983.r153703

**Published:** 2026-05-18

**Authors:** Jun Kim

**Affiliations:** 1 Chungnam National University, Daejeon, Daejeon, South Korea

This report presents a high-quality genome assembly of Heligmosomum mixtum. As the Tree of Life team has already established a highly reliable workflow for data generation and analysis, I do not have any major comments regarding the analytical procedures themselves. I would, however, like to ask the authors to consider the following two points.

 “Most of the assembly sequence (98.33%) was assigned to 6 chromosomal-level scaffolds, representing 5 autosomes and the X sex chromosome.”

 --> I would appreciate it if the authors could examine the telomeric repeats at the ends of the chromosomes. In addition, the read depth of the X sex chromosome should also be evaluated. My understanding is that this species probably has an XX/XO sex determination system, and it would be helpful if this point could be described in the manuscript. If the XX/XO system is indeed correct, the read depth of the X chromosome would be expected to be approximately half that of the autosomes, so confirmation of this would strengthen the study. Although the use of whole-genome amplification likely results in uneven read depth, it would still be informative to assess whether the X chromosome shows roughly half the coverage of the autosomes and whether there are any regions with unusually elevated read depth.

 “For haplotype 1, the estimated QV is 58.2, and for haplotype 2, 57.0. When the two haplotypes are combined, the assembly achieves an estimated QV of 57.5. The k-mer completeness is 88.23% for haplotype 1, 73.52% for haplotype 2, and 99.23% for the combined haplotypes (Figure 4).”

 --> It appears that only HiFi data were generated. If that is the case, it may be worth noting that the HiFi reads used as input for QV estimation with MerquryFK were likely the same HiFi reads used for the genome assembly itself, and that this may have led to an overestimation of the QV.

Are sufficient details of methods and materials provided to allow replication by others?

Yes

Is the rationale for creating the dataset(s) clearly described?

Yes

Are the datasets clearly presented in a useable and accessible format?

Yes

Are the protocols appropriate and is the work technically sound?

Yes

Reviewer Expertise:

Genome assembly

I confirm that I have read this submission and believe that I have an appropriate level of expertise to confirm that it is of an acceptable scientific standard.

# Reviewer report: Reviewer response for version 1

**DOI:** 10.21956/wellcomeopenres.28983.r153704

**Published:** 2026-05-13

**Authors:** James Wasmuth

**Affiliations:** 1 University of Calgary, Calgary, Canada

This is a solid and useful genome resource for *Heligmosomum mixtum*, a species with clear relevance for parasitology, eco-immunology, and comparative nematode genomics. The manuscript is clearly written, and the rationale for generating the dataset is well articulated. Methods are described with sufficient detail to support reproducibility, and the combination of PacBio HiFi data, Hi-C scaffolding, and manual curation has resulted in a high-quality chromosome-level assembly.

 The assembly metrics are strong. The haplotype-resolved assembly is also a strength, particularly for a wild-derived parasite where heterozygosity is expected to be biologically meaningful.

 One point to note is that BUSCO completeness (~92%) suggests there is still a fraction of conserved gene content that is either missing or fragmented. This is not unusual, but it does indicate some remaining scope for improvement, whether through further polishing or downstream annotation.

Are sufficient details of methods and materials provided to allow replication by others?

Yes

Is the rationale for creating the dataset(s) clearly described?

Yes

Are the datasets clearly presented in a useable and accessible format?

Yes

Are the protocols appropriate and is the work technically sound?

Yes

Reviewer Expertise:

Nematode genomics

I confirm that I have read this submission and believe that I have an appropriate level of expertise to confirm that it is of an acceptable scientific standard.

# Reviewer report: Reviewer response for version 1

**DOI:** 10.21956/wellcomeopenres.28983.r153709

**Published:** 2026-05-05

**Authors:** Erich M. Schwarz

**Affiliations:** 1 Cornell University, Ithaca, New York, USA

This genome assembly is done to the usual high Tree of Life standard and leaves little to comment upon: technically, and in the clarity of its writing, and in its selection of a useful resource for comparative genomics of model gastrointestinal nematode parasites, it is pleasing to read.  The remaining analysis that will need to be done by other people using this is protein-coding gene prediction, but improvements in software make this an increasingly easy deficit to remedy.

Are sufficient details of methods and materials provided to allow replication by others?

Yes

Is the rationale for creating the dataset(s) clearly described?

Yes

Are the datasets clearly presented in a useable and accessible format?

Yes

Are the protocols appropriate and is the work technically sound?

Yes

Reviewer Expertise:

Nematode genomics, in both non-parasitic and parasitic nematode species

I confirm that I have read this submission and believe that I have an appropriate level of expertise to confirm that it is of an acceptable scientific standard.
